# A case of recto-sigmoid endometriosis mimicking carcinoma

**DOI:** 10.1186/s40064-016-2221-6

**Published:** 2016-05-17

**Authors:** Ritu Rana, Sandeep Sharma, Harjeet Narula, Brijesh Madhok

**Affiliations:** Mid Yorkshire Hospitals NHS Trust, Wakefield, UK

## Abstract

**Introduction:**

Although endometriosis with sigmoid serosal involvement is not uncommon in women of childbearing age, the mucosal and lymph node involvement is rare and differential diagnosis from colon cancer and diverticulitis may be difficult due to poor diagnostic accuracy of colonoscopy and colonic biopsies.

**Case presentation:**

We present a case of a nulliparous woman presenting with large bowel obstruction. She underwent emergency sigmoid colectomy based on clinical and radiological findings. At operation, the pathology was thought to be primary sigmoid tumour. However, histopathological examination of the sigmoid colon led to the final diagnosis of large intestinal endometriosis.

**Conclusion:**

Rectosigmoid endometriosis is often difficult to diagnose but should be considered in differential diagnosis of child bearing aged women with lower gastrointestinal tract obstruction.

## Background

Endometriosis is characterized by the presence of functional endometrial tissue consisting of glands and/or stroma located outside the uterus (Olive and Schwartz 1993). Its incidence is 5–15 % in menstruating women (Olive and Schwartz 1993; Lu and Ory 1995; Keane and Peel 1990).

GI involvement of endometriosis has been found in 3.8–37 % of women diagnosed with endometriosis.

We report a case in which endometrial infiltration of the sigmoid colon caused acute intestinal obstruction necessitating emergency laparotomy. Diagnosis of recto sigmoid endometriosis was only made by pathological examination of the resected specimen.

## Case report

A 43-year-old Caucasian nulliparous woman presented to Accident and Emergency with 6 days history of episodic abdominal pain and vomiting.

There was no previous medical or surgical history. Patient had 27–28-day menstrual cycles and menstrual periods lasted 6–7 days with normal blood loss. She had no documented family history of any major medical or surgical problems. She was nulliparous by choice.

On examination, her abdomen was soft, minimally tender all over, with slight distension. All blood test results were normal. Abdominal X-ray revealed dilated small and large bowel. Differential diagnoses were thought to be either diverticular disease causing stricture or intestinal obstruction secondary to band adhesion.

**Fig. 1:**
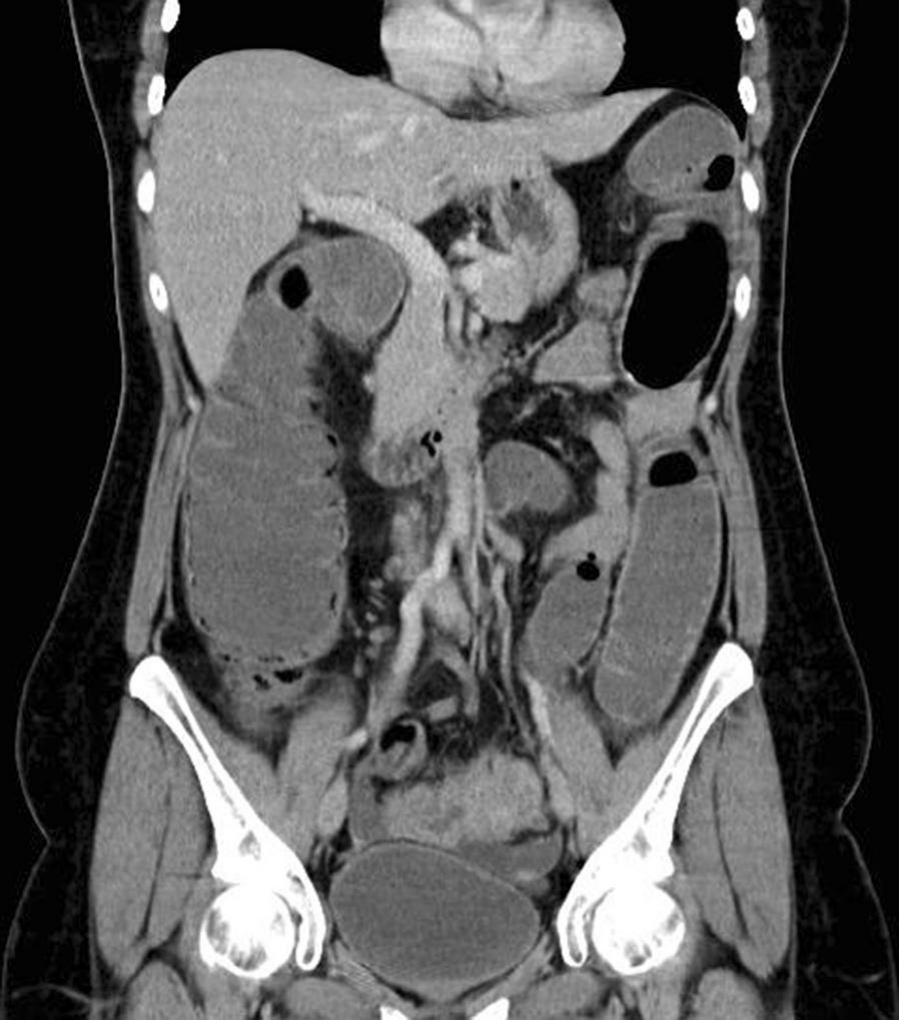
CT images demonstrating thick sigmoid colon with large bowel obstruction and left ovarian cyst

**Fig. 2:**
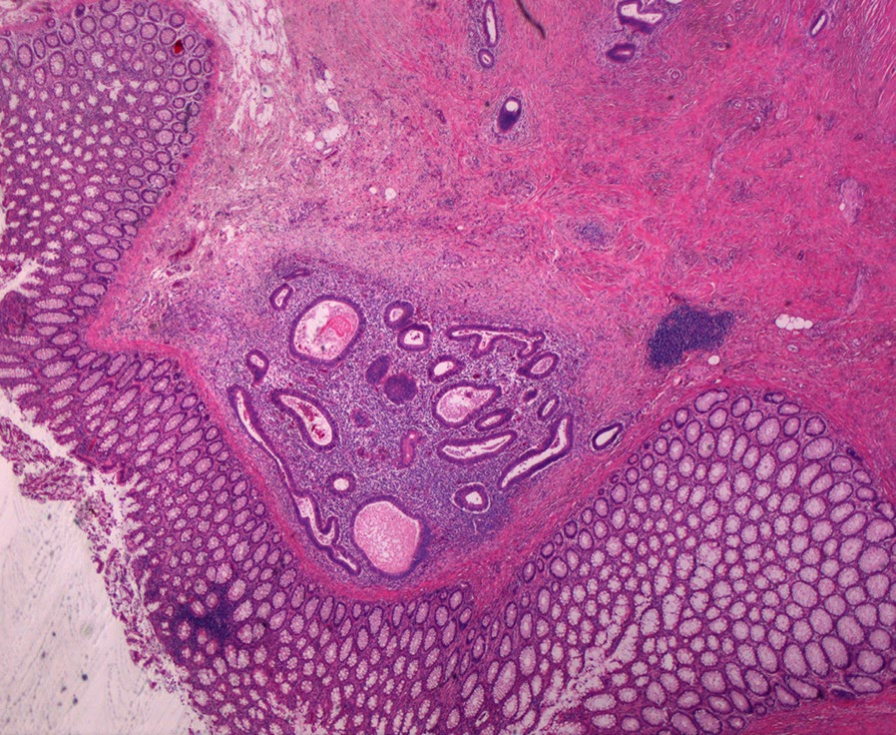
Haematoxylin eosin staining demonstrates sigmoid colon with deeply infiltrating Endometriosis

She underwent a CT scan abdomen and pelvis, which revealed dilated small and large bowel loops with, thickened sigmoid colon. The diagnosis was thought to be primary colorectal cancer, particularly as there were enlarged lymph nodes visualised on CT scan. Ultrasound Scan of pelvis showed left ovarian simple cyst and right ovary with a complex cyst due to low level echoes and focal wall nodularity. Flexible sigmoidoscopy confirmed an obstruction at the level of sigmoid colon. Emergency laparotomy was undertaken that found a recto-sigmoid tumour causing intestinal obstruction. There was no evidence of distant metastasis. Oncological resection of the tumour was carried out and Hartmann’s procedure was performed. Resected sigmoid colon with lymph nodes was sent to histology. Both ovaries were enlarged and adherent posteriorly and to pelvic side wall. She was reviewed intra operatively by the on call Gynaecologist, who arranged for tumour markers and a pelvic Ultrasound Scan. Left ovary had a 40.9 mm simple cyst, right ovary contained a complex cyst measuring 24 × 43 × 37 mm, there was evidence of hydrosalpinx on the right side. CA125 was 61 Ku/L with a CA 199 of 52 Ku/L. Histology revealed no evidence of malignancy, but a deeply infiltrating endometriosis involving the bowel and lymph nodes. Of the 21 lymph nodes harvested, 6 showed involvement with endometriosis. These results were discussed at the Gynaecology multi disciplinary team meeting and decision was made to start patient on GnRH agonist for treatment of endometriosis followed by abdominal hysterectomy and bilateral salpingo-oophorectomy at the same time when surgeons plan a reversal of her colostomy (Figs. 1, 2).

Patient had her first Gynaecological consultation after all relevant investigation and Multi Disciplinary Team meeting. She was relieved to find out that there was no bowel cancer. She was certain that she did not want to have any children. In view of her extensive endometriosis she was treated with GnRH analogue injections for 4 months, extended to 8 months due to delay in surgery. She had a hysterectomy with removal of both ovaries, excision of endometriosis with reversal of the Hartmann’s procedure jointly with the colorectal team. Surgery was uncomplicated and she made a good postoperative recovery.

## Discussion

Bowels are the most common site of extra pelvic endometriosis affecting 3.8–37 % of women with endometriosis. The most common sites are sigmoid colon and rectum followed by the ileum, appendix and cecum (Remorgida et al. 2007).

Superficial nodules are more common involving bowel serosa and the muscularis propria. Rarely the lesions are deep seated reaching the mucosal layers. Common presentation of intestinal endometriosis is with dyschezia, abdominal bloating, diarrhoea, constipation, cyclical rectal bleeding. Deeper lesions may cause fibrosis, thickening of bowel mucosa leading to stenosis which may present with bowel obstructions. Very rarely bowel endometriosis may cause perforation or undergo malignant transformation.

The clinical presentation and radiological appearance of bowel endometriosis may be confused with irritable bowel syndrome, inflammatory bowel disease, colitis, diverticular disease or neoplasm. The gold standard test for diagnosis of pelvic endometriosis is laparoscopy with histological confirmation of endometriosis. Bowel lesions can be seen on surface of bowel during laparoscopy without diagnosing the depth of these nodules. Bowel endometriosis may be palpated as recto-vaginal nodules on a thorough pelvic examination under anaesthesia. The exact depth of infiltration of bowel endometriosis and the degree of luminal obstruction, if any, can be fairly accurately diagnosed by a pelvic MRI scan. CT scan and barium enema are good tests to demonstrate bowel compression, stenosis or filling defect. Several studies have also demonstrated successful use of transvaginal ultrasonography and rectal endoscopic ultrasound in diagnosing the presence and estimating the depth of recto-sigmoid endometriosis. This technique is however dependent on operator experience.

Colonoscopy is a good test to rule out colorectal cancer. It has very limited diagnostic value in recto-sigmoid endometriosis as most of these lesions do not involve the mucosal layer and even if the mucosa is involved, the endoscopic appearance is not diagnostic. Biopsy usually does not provide adequate tissue for diagnosis of endometriosis.

Treatment of bowel endometriosis should be based on site and size of the endometriotic nodule, presenting symptoms of patient and their fertility desires. Treatment can be medical or surgical. Medical treatment involves hormonal therapy including combined oral contraceptives, progestagens and anti-progestagens, GnRH-agonists and antagonists and aromatase inhibitors. The side effects of each therapy needs to be discussed in detail with patient (ESHRE guideline: management of women with endometriosis). Hormonal therapies may improve symptoms but do not prevent the progression of intestinal endometriosis. Surgical treatment involves either shaving of nodules from the bowel surface or segmental resection. Since segmental resection is associated with higher morbidity, laparoscopic nodulectomy is being commonly practised for lesions that have not caused significant stenosis of bowel. This is associated with fewer intra-operative and post-operative complications along with comparable symptom relief.

Our patient represents a case of symptomatic gastrointestinal endometriosis with mucosal involvement, without a previous history of pelvic endometriosis.

In conclusion, acute bowel obstruction secondary to intestinal endometriosis is often a diagnostic challenge mimicking a broad spectrum of diseases. Endometriosis as a diagnosis should be considered in any young woman with symptoms from the lower gastrointestinal tract.
